# Genome Sequences of Cluster A Mycobacteriophages Dorothea and LappelDuVide, Isolated on *Mycobacterium smegmatis* mc ^2^ 155

**DOI:** 10.17912/micropub.biology.001881

**Published:** 2025-11-18

**Authors:** Nicole Pasterczyk, Claire Wilson, Zoe Broker, Audrey Chobor, DeAndrea Daughtry, Odinakachukwu Dibor, Annly John, Sruthvika Kandru, Mokhinur Kodirova, Constance Mulligan, Chelse Owate, Olivia Pistone, Sophia Smith, Sarah Teitelman, Samantha Victor, Petra Oganovich, Madelyn Hult, Rhema Hooper, Ellie Novak, Sameen Mariaca, Lee Graham, Stephen Mensah, Margaret Kenna, Vassie Ware

**Affiliations:** 1 Biological Sciences, Lehigh University, Bethlehem, PA USA

## Abstract

Genome annotation of *Mycobacterium smegmatis* phages Dorothea and LappelDuVide revealed genomic features/organization common to cluster A mycobacteriophages; they are assigned to subclusters A6 and A4, respectively. Both genomes encode a putative immunity repressor, but the mode of prophage inheritance likely differs; Dorothea encodes a ParA/B partitioning system while LappelDuVide encodes a serine integrase. Dorothea encodes 3 tRNAs while none were identified for LappelDuVide.

**Figure 1. Dorothea and LappelDuVide: Phage Morphologies and Partial Genome Comparisons:**
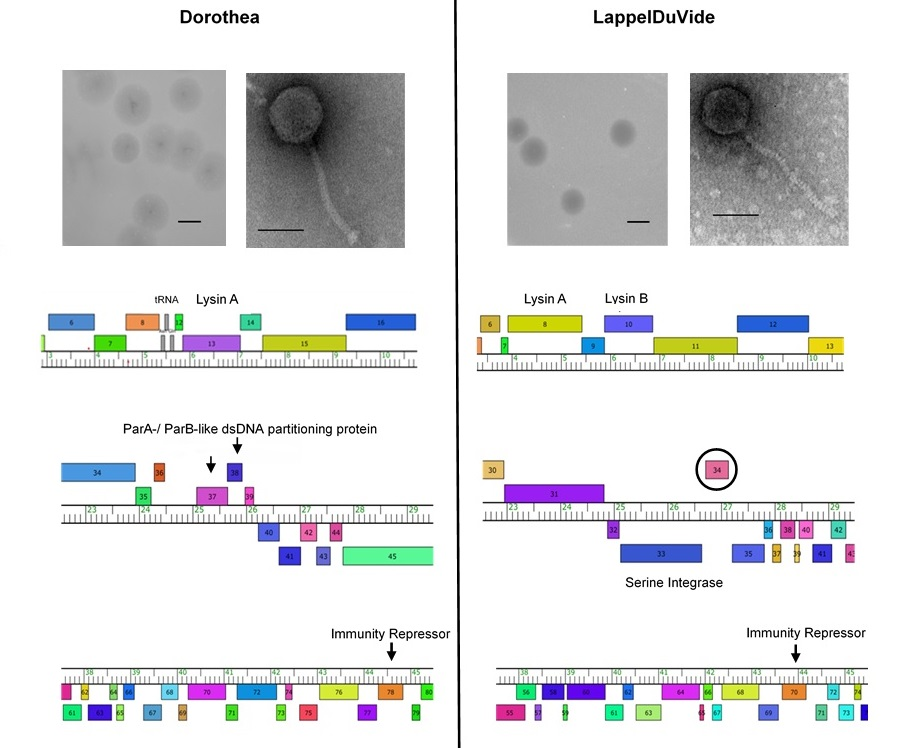
Dorothea, top left; LappelDuVide, top right. Plaque images: scale bar is 5mm. Plaques with clear centers and turbid halos are visible. TEM images: scale bar is 50nm. TEM images show siphoviral morphologies. Phamerator maps: Dorothea, bottom left; LappelDuVide, bottom right. Three distinct regions of each genome are shown: genome coordinates are 3000-10,500 bp (Region 1); 22,500-29,500 bp (Region 2); and 37,500-45,500 bp (Region 3), respectively. Region 1 shows tRNAs (3 gray boxes) and lysins. Region 2 includes the gene encoding the serine integrase (on the reverse strand) in LappelDuVide and genes encoding ParA (purple box with arrow)/ParB (blue box with arrow) dsDNA partitioning proteins on the forward strand in Dorothea. The gene (34) encircled in black on the forward strand in LappelDuVide disrupts the typical transcriptional direction on the right arm. Region 3 shows the gene encoding the immunity repressor, found distal to machinery involved in lysogeny establishment in both phages. Protein coding genes are depicted with colored boxes.

## Description

Given the contributions of bacteriophage research to unravelling fundamentals of molecular biology and developing novel strategies to combat bacterial antibiotic resistance, continued discovery of new bacteriophages and analysis of their unique features is critical to sustain advances in these areas (Haq et al., 2012). Cluster A mycobacteriophages are the most abundant phage group catalogued in the Actinobacteriophage Database (Russell and Hatfull, 2017), sharing many common genetic features and genomic architecture, yet presenting with a high degree of genetic diversity between subclusters (Hatfull, 2012).

Mycobacteriophage LappelDuVide was isolated from direct extraction of soil (Hatfield, PA, USA). Mycobacteriophage Dorothea was isolated from an enriched soil sample (Bethlehem, PA, USA). Briefly for direct isolation, soil samples were suspended in phage buffer with shaking for 1 hour, filtered (0.2 micron pore size), and the filtrate plated in top agar with *Mycobacterium smegmatis* mc ^2^ 155. Plates were incubated at 37 ˚C for 2 days. For enriched isolation, the soil and *M. smegmatis* were suspended in 7H9 liquid medium and incubated with shaking for 3 days at 37 ˚C before the resulting culture was filtered and the filtrate plated (Zorawik et al., 2024). LappelDuVide was purified after 4 rounds of plaque assays; Dorothea was purified after 6 rounds. Plaque morphology characteristics are shown (Table 1, Fig.1). High titer lysates, used in negative-stain transmission electron microscopy (TEM) preparations, revealed siphovirus morphologies for both phages (Table 1, Fig.1).

**Table 1: Phage Characterization**

**Table:** 

	**Dorothea (A6)**	**LappelDuVide (A4)**
Plaque Morphology	Clear center, turbid halo	Clear center, turbid halo
Plaque size @ 24hrs (mm)	5 (n=10)	3-5 (n=10)
Phage morphology	Siphovirus	Siphovirus
Capsid width (nm)	50 +/-1 (n=5)	50 +/-1 (n=5)
Tail length (nm)	100 +/-1 (n=5)	125 +/-1 (n=5)
Capsid width : Tail length ratio	1:2	1:2.5
Genome Size (bp)	52,637	51,371
# Raw (150 bp single-end) reads; [shotgun coverage]	319,931 [868]	80,883 [208]
Genome terminus (3' sticky overhang)	5' CGGTCGGTTA 3'	5' CGGCCGGTAA 3'
GC content (%)	61.6	63.9

LappelDuVide DNA was isolated from lysate by column purification using a Promega Wizard DNA Clean-up Kit. Dorothea DNA was isolated from lysate using a standard phenol/chloroform extraction method (Mülhardt and Beese, 2007). Genomes were prepared for sequencing with an NEB Ultra II FS Library Kit and sequenced on an Illumina MiSeq 1000 (v3 reagents). The resulting 150 base reads were assembled without any trimming or QC. Raw reads were assembled using Newbler version 2.9 (Miller et al., 2010) and Consed version 29 (Gordon and Green, 2013) into a single contig (Table 1). Genomic termini were determined as previously described (Russell, 2018).

Genomes were annotated using DNA Master v5.23.6 (Lawrence, 2007), GeneMark v2.5 (Bessmer and Borodovsky, 2005), Glimmer v3.02 (Delcher et al., 2007), and Starterator v.546 (Pacey, 2016). PECAAN (Rinehart et al., 2016)) was also used for LappelDuVide annotation. BLAST, using the Actinobacteriophage (Russell and Hatfull, 2017) and NCBI non-redundant databases (Altschul et al., 1990) and HHPRED, using the PDB_mmCIF70, Pfam- v.36, NCBI Conserved Domains databases v57v87 (Zimmermann et al., 2018) were used for functional annotation. Phamerator, using Actino_draft database v578 (Cresawn et al., 2011), assessed synteny. tRNAs were identified using tRNAscan v2.0.6 (Lowe and Eddy, 1997) and ARAGORN v.1.2.38 (Laslett and Canback, 2004). TMHMM v1.0.24 (Möller et al., 2001) predicted transmembrane domains. Default settings were used for all software.

The Dorothea genome includes 99 predicted protein-encoding genes and three tRNAs: tRNA-Asn, tRNA-Trp, and tRNA-Gln ( Fig. 1, Region 1). The LappelDuVide genome has 88 predicted protein-encoding genes and no tRNAs. In general for both phages, a contiguous segment of the genome on the left arm includes the majority of the structural and assembly genes transcribed in one direction from left to right on the forward strand. The remaining genes on the right arm in Dorothea are transcribed in the opposite direction, on the reverse strand. This is also generally true for LappelDuVide, except for one gene ( *34* ) positioned just upstream of the serine integrase (located on the reverse strand) ( Fig. 1, Region 2). Both genomes encode an immunity repressor in the same pham (identified as a group of proteins with a high degree of amino acid similarity to one another), located in the same relative syntenic position on the reverse strand on the right arm (Cresawn et al., 2011) ( Fig. 1, Region 3). Notable differences between the genomes include the presence of lysins A and B in LappelDuVide, but only lysin A in Dorothea. By genome analysis, both phages should be capable of lysogen establishment, but lysogeny would likely occur by different mechanisms. Dorothea has a ParA/ParB partitioning system (Dedrick et al., 2016) on the forward strand and LappelDuVide has a serine integrase (Smith, 2015) ( Fig. 1, Region 2).

**Nucleotide sequence accession numbers** : Dorothea is available at GenBank with Accession No. PQ184798 and Sequence Read Archive (SRA) No. SRX26413417 . LappelDuVide is available at GenBank with Accession No. PP978813 and SRA No. SRX26413423 .

## References

[R1] Altschul Stephen F., Gish Warren, Miller Webb, Myers Eugene W., Lipman David J. (1990). Basic local alignment search tool. Journal of Molecular Biology.

[R2] Besemer J., Borodovsky M. (2005). GeneMark: web software for gene finding in prokaryotes, eukaryotes and viruses. Nucleic Acids Research.

[R3] Cresawn Steven G, Bogel Matt, Day Nathan, Jacobs-Sera Deborah, Hendrix Roger W, Hatfull Graham F (2011). Phamerator: a bioinformatic tool for comparative bacteriophage genomics. BMC Bioinformatics.

[R4] Dedrick Rebekah M., Mavrich Travis N., Ng Wei L., Cervantes Reyes Juan C., Olm Matthew R., Rush Rachael E., Jacobs‐Sera Deborah, Russell Daniel A., Hatfull Graham F. (2016). Function, expression, specificity, diversity and incompatibility of actinobacteriophage
*parABS*
systems. Molecular Microbiology.

[R5] Delcher Arthur L., Bratke Kirsten A., Powers Edwin C., Salzberg Steven L. (2007). Identifying bacterial genes and endosymbiont DNA with Glimmer. Bioinformatics.

[R6] Gordon David, Green Phil (2013). *Consed:*
a graphical editor for next-generation sequencing. Bioinformatics.

[R7] Haq Irshad Ul, Chaudhry Waqas Nasir, Akhtar Maha Nadeem, Andleeb Saadia, Qadri Ishtiaq (2012). Bacteriophages and their implications on future biotechnology: a review. Virology Journal.

[R8] Hatfull Graham F. (2012). The Secret Lives of Mycobacteriophages. Advances in Virus Research.

[R9] Laslett D. (2004). ARAGORN, a program to detect tRNA genes and tmRNA genes in nucleotide sequences. Nucleic Acids Research.

[R10] Lawrence, J. DNAMaster. (2007). Retrieved from http://cobamide2.bio.pitt.edu/computer.htm

[R11] Lowe Todd M., Eddy Sean R. (1997). tRNAscan-SE: A Program for Improved Detection of Transfer RNA Genes in Genomic Sequence. Nucleic Acids Research.

[R12] Miller Jason R., Koren Sergey, Sutton Granger (2010). Assembly algorithms for next-generation sequencing data. Genomics.

[R13] Möller Steffen, Croning Michael D. R., Apweiler Rolf (2001). Evaluation of methods for the prediction of membrane spanning
regions. Bioinformatics.

[R14] (2007). Molecular Biology and Genomics.

[R15] Pacey, M. (2016). Starterator guide. University of Pittsburgh, Pittsburgh, PA.

[R16] Reinehart, C., Gaffney, B., Wood, J. D., & Smith, J. (2016). PECAAN, a Phage Evidence Collection And Annotation Network. https://discover.kbrinsgd.org

[R17] Russell, D. A., & Hatfull, G. F. (2017). PhagesDB: The actinobacteriophage database. Bioinformatics, 33(6), 784–786. 10.1093/bioinformatics/btw711 PMC5860397 28365761

[R18] Russell Daniel A. (2017). Sequencing, Assembling, and Finishing Complete Bacteriophage Genomes. Methods in Molecular Biology.

[R19] Smith Margaret C. M. (2015). Phage-encoded Serine Integrases and Other Large Serine Recombinases. Microbiology Spectrum.

[R20] Zimmermann Lukas, Stephens Andrew, Nam Seung-Zin, Rau David, Kübler Jonas, Lozajic Marko, Gabler Felix, Söding Johannes, Lupas Andrei N., Alva Vikram (2018). A Completely Reimplemented MPI Bioinformatics Toolkit with a New HHpred Server at its Core. Journal of Molecular Biology.

[R21] Zorawik Michelle, Jacobs-Sera Deborah, Freise Amanda C., Reddi Krisanavane, SEA-PHAGES (2024). Isolation of Bacteriophages on Actinobacteria Hosts. Methods in Molecular Biology.

